# Antiparasitic Activity of *Hippeastrum* Species and Synergistic Interaction between Montanine and Benznidazole against *Trypanosoma cruzi*

**DOI:** 10.3390/microorganisms11010144

**Published:** 2023-01-06

**Authors:** Mauricio Piñeiro, Javier E. Ortiz, Renata M. Spina Zapata, Patricia A. Barrera, Miguel A. Sosa, Germán Roitman, Jaume Bastida, Gabriela E. Feresin

**Affiliations:** 1 Instituto de Biotecnología, Facultad de Ingeniería, Universidad Nacional de San Juan, Av. Libertador General San Martin 1109 O, San Juan CP 5400, Argentina; 2 Consejo Nacional de Investigaciones Científicas y Técnicas (CONICET), Ciudad Autónoma de Buenos Aires (CABA), Godoy Cruz CP 2290, Argentina; 3 Facultad de Ciencias Médicas, Instituto de Histología y Embriología “Dr. Mario H. Burgos”, Universidad Nacional de Cuyo-CONICET, Mendoza CP 5500, Argentina; 4 Facultad de Turismo y Urbanismo, Universidad Nacional de San Luis, Av. del Libertador San Martín 721 Villa de Merlo, San Luis CP D5881DFN, Argentina; 5 Departament de Biologia, Sanitat i Medi Ambient, Facultat de Farmàcia i Ciències de l’Alimentació, Universitat de Barcelona, 08028 Barcelona, Spain

**Keywords:** Amaryllidaceae, Chagas disease, alkaloids, neglected tropical diseases

## Abstract

Background: *Hippeastrum* species have a wide range of biological properties. In Argentina, this genus comprises ten widely distributed species. Purpose: To evaluate the antiparasitic and anticholinesterase activities and chemical profiles of seven Argentinean *Hippeastrum* species and determine the synergism between the major isolated alkaloid—montanine—and benznidazole in anti-*Trypanosoma cruzi* activity. Methods: The antiparasitic activity was evaluated through antiproliferative and viability assays against *T. cruzi* epimastigotes. Synergism assays were performed using the Chou–Talalay method. AChE and BuChE inhibitory activities were also assessed. The alkaloid composition was obtained using GC-MS analysis. Results: All extracts showed strong growth inhibition of *T. cruzi* epimastigote proliferation. The extracts from *H. aglaiae*, *H. aulicum*, and *H. hybrid* stand out for their potent and total growth inhibition, which was comparable to benznidazole. The *H. reticulatum* extract showed strong Acetylcholinesterase (AChE) inhibitory activities, while five species showed moderate Butyrylcholinesterase (BuChE) inhibition. Fifteen alkaloids were identified by means of GC-MS. Regarding the synergism assessment, the highest synergistic effect was obtained from the combination of montanine and benznidazole. Conclusion: *Hippeastrum* species bulb extracts from Argentina were shown to be a good source of antiparasitic alkaloids and cholinesterase inhibitors. The synergism between montanine and benznidazole emerges as a potential combination for future studies to treat Chagas disease.

## 1. Introduction

Natural products have been a source of compounds useful in medicine, pharmaceuticals, and biology. Neglected Tropical Diseases (NTDs) are a diverse group of communicable diseases, and they are prevalent in tropical and subtropical conditions and affect more than 1 billion people [1]. WHO lists 17 NTDs, including Chagas disease (CD), which is caused by the protozoan parasite *Trypanosoma cruzi*. CD is mainly found in endemic areas of 21 countries in Latin America, and it was estimated that in the world, there are between 6 and 7 million infected people [1]. The drugs nifurtimox and benznidazole (Bzn) are effective against *T. cruzi* during the acute phase of the disease but are less useful in the chronic phase [2]. These drugs cause frequent adverse effects, such as allergic skin reactions, rashes, nerve damage (peripheral neuropathy), and diminished bone marrow function (neutropenia and thrombocytopenia). Limitations especially arise during prolonged treatment if the patient discontinues treatment [3,4]. Numerous synthetic and natural compounds were tested against *T. cruzi* for decades, but in most cases, their use is restricted by their high cytotoxicity and low efficacy in the chronic phase of CD [5,6]. Considering all of the disadvantages of current antichagasic chemotherapy, alternative drugs with safer, more tolerable, and effective profiles remain a critical need. Moreover, there is an increasing interest in drug combinations for Chagas disease, allowing a reduction in the dose of each constituent and a consequent reduction in adverse effects and treatment withdrawal [7]. In fact, the majority of cancer chemotherapies and several antimicrobial treatments are based on multidrug regimens [8]. On the other hand, 50 million people around the world live with dementia [9]. Alzheimer’s disease (AD) is the most common dementia type and may account for 60–70% of dementia cases [10,11].

Alkaloids are secondary metabolites of plants that are of great interest for drug development [12]. A particular characteristic of the Amaryllidaceae family plants is an exclusive, large, and still-expanding group of alkaloids that are characterized by a unique skeletal arrangement and a broad spectrum of biological activities [12,13,14]. In northeastern Argentina, indigenous communities use some species of the Amaryllidaceae family in traditional medicine, including *Hippeastrum* species [15]. The genus *Hippeastrum*, belonging to the Amaryllidoideae subfamily, is endemic to South America and is characterized by large bulbs and prominent and colorful flowers [16]. *Hippeastrum* species have a wide range of biological properties, including antimicrobial, cholinesterase inhibitory, cytotoxic, psychoactive, antiepileptic, and anti-inflammatory activities [16,17,18,19,20]. In Argentina, this genus comprises nine widely distributed and poorly studied species that vary in habitat, ranging from tropical to subtropical areas and from sea level to high altitudes [21,22].

In this work, an in vitro study of basic alkaloid-rich extracts (BAREs) from *Hippeastrum* spp. was performed to determine their antiparasitic activities, as well as to estimate their potential as cholinesterase inhibitors. Furthermore, the alkaloid profile of each species using GC-MS was obtained. Finally, the combination of the isolated montanine alkaloid, *H. hybrid* BARE, and Bzn was tested to determine their synergistic effect against *T. cruzi*.

## 2. Materials and Methods

### 2.1. Plant Material

Bulbs of the species *Hippeastrum aglaiae*, *H. aulicum*, *H. glaucescens*, *H. hybrid*, *H. petiolatum*, *H. puniceum*, *and H. reticulatum* were collected in Northeast Argentina during the flowering period between the years 2012 and 2015. The specimens were identified by MSc. German Roitman and then deposited in the herbarium of Universidad de Nacional de San Juan under specific herbarium codes as follows: IBT-Arg 20, IBT-Arg 21, IBT-Arg 22, IBT-Arg 23, IBT-Arg 24, IBT-Arg 25, and IBT-Arg 26. 

### 2.2. Alkaloid Extraction and Isolation

Dry powdered bulb material (20–100 g) was macerated in H_2_SO_4_ 2% for 4 h in an ultrasonic bath (3 × 1000 mL). Subsequently, samples were centrifuged at 4200× *g* (10 min), and the supernatant was transferred to another flask, where it was defatted with diethyl ether (Et_2_O) (3 × 500 mL). The aqueous solution was brought to pH 10–11 with 10% NaOH, and the alkaloids were extracted with CH_2_Cl_2_ (3 × 500 mL). The organic phase was dried with anhydrous sodium sulfate and then evaporated to obtain the basic alkaloid-rich extracts (BAREs) of each species. The average alkaloid yield of the extraction process was 0.55% from the dry plant material. Montanine was previously isolated from the bulbs of *Hippeastrum argentinum* [17]. The *H. hybrid* BARE was chromatographed on a Sephadex LH-20 column (length 50 cm, 3.5 cm i.d.; equilibrated to MeOH) and then eluted with MeOH. Twenty-one fractions were obtained and grouped according to their TLC profiles as follows: fractions 1 to 10 (F1), fractions 11 and 12 (F2), fractions 13 and 14 (F3), fractions 15 and 16 (F4), fractions 17 to 19 (F5), and fractions 20 and 21 (F6). As a result of the isolation process, six fractions were obtained (F1–F6).

### 2.3. GC-MS and UHPLC-MS/MS Analysis

The alkaloids were identified by comparing their GC-MS spectra and Kovats retention index (RI) values against authentic Amaryllidaceae alkaloids previously isolated and identified. Spectral data were processed with AMDIS 2.64 software. Alkaloids were identified by comparing their fragmentation patterns and RIs with those of the Amaryllidaceae alkaloids in our laboratory library, in which the isolated compounds were identified by NMR and other spectroscopic techniques (UV, CD, and MS), as well as the NIST database and literature data. The RI values were calibrated with an *n*-hydrocarbon calibration mixture (C9–C36). The results obtained were analyzed using AMDIS 2.64 software and the NIST database. Chromatograms indicating the identified alkaloids are shown in Supplementary Materials (Figure S1).

The UHPLC-MS/MS quantification analyses were performed on a Waters Acquity H-class with a Xevo TQ-S micro mass spectrometer detector, and the MassLynx software was used for data acquisition and treatment. An Acquity UPLC BEH C18, 130 Å, 1.7 µm, 2.1 mm × 100 mm column was used. The elution system was composed of formic acid 0.1% (mobile phase A), acetonitrile–formic acid 0.1% (mobile phase B), and MeOH (mobile phase C). The flow rate was 0.2 mL·min^−1^, while the temperature of the sample compartment and column were 22 °C and 40 °C, respectively. The gradient started with 95% A and 5% B (hold for 2 min); 85% A and 15% B (3 min); 80% A, 10% B, and 10% C (5 min) and hold for 7 min; 95% A and 5% B (1 min) and hold for 2 min, totaling 20 min of analysis for montanine quantification. The spectrometric parameters were ESI + and the daughter ion function for the [M + H]^+^ of montanine (*m*/*z* 302), with a voltage cone of 46.15 V, capillary energy of 2.00 kV, source temperature of 124 °C, and collision energy of 20 V. Standard solutions of montanine for the calibration curve (0.1, 1, 5, and 10 ppm), as well as the BARE solutions (84, 96, and 98 ppm for *H. hybrid*, *H. aglaiae*, and *H. puniceum*, respectively), were prepared by dissolving them in a mixture of methanol and water (50:50). The solutions were filtered through a 0.22 μm membrane filter, and the injection volume was 10 μL.

### 2.4. AChE and BuChE Inhibitory Activities

Cholinesterase inhibitory activities were determined according to Ellman et al. [23] with some modifications [16]. Acetylcholinesterase (AChE) from *Electrophorus electricus* (C3389), Butyrylcholinesterase (BuChE) from equine serum (C7512), acetylthiocoline iodide (ATCI, A5751), butyrylthiocholine iodide (BTCI, 20820), 5,5′-dithio-bis (2-nitrobenzoic acid) (DTNB) (D-8130), and galantamine hydrobromide (GAL) were purchased from Sigma-Aldrich (St. Louis, MO, USA. A volume of 50 μL of 0.25 U/mL AChE or BuChE in phosphate buffer (8 mM K_2_HPO_4_, 2.3 mM NaH_2_PO_4_, and 0.15 M NaCl, pH 7.6) and 50 μL of each BARE concentration dissolved in the same buffer were added to the wells. The plates were incubated for 30 min at room temperature before 100 μL of the substrate solution (0.1 M Na_2_HPO_4_, 0.5 M DTNB, and 0.6 mM ATCI or BTCI in Millipore water, pH 7.5) was added. The absorbance was read in a Thermo Scientific Multiskan FC microplate spectrophotometer at 405 nm after 5 min. The enzyme-inhibitory activity was calculated as a percentage compared to an assay using a buffer without any inhibitor. The enzyme-inhibitory data were analyzed with the software package Prism (Graph Pad Inc., San Diego, CA, USA). The BARE concentrations used to calculate the IC_50_ values were 1, 20, 40, 60, 80, and 100 μg/mL in both AChE and BuChE assays. The IC_50_ values are the means ± SD of three individual determinations, each performed in triplicate.

### 2.5. Trypanosoma cruzi 

#### 2.5.1. Culture

*T. cruzi* epimastigotes (Dm28c strain—DTU: TcI) were cultured at 28 °C in Diamond medium (0.1 M NaCl, 0.05 M K_2_HPO_4_, 0.625% (*w*/*v*) tryptose, 0.625% (*w*/*v*) tryptone, and 0.625% (*w*/*v*) yeast extract, pH 7.2), supplemented with 10% inactivated FBS (Gibco) and 12.5 µg/mL hemin and antibiotics 0.1% (penicillin 75 U/mL and streptomycin 75 µg/mL).

#### 2.5.2. Growth Inhibition Assay

Parasites were incubated at 28 °C in sterile plastic tubes with 10 and 50 µg/mL of each BARE; 1, 2.5, 5, and 10 µg/mL of *H. aglaiae* and *H. hybrid* BAREs; 5 and 10 µg/mL of each fraction of *H. hybrid* BARE; and 0.1, 0.5, 1, 2.5, and 5 µg/mL of montanine. The initial concentration (InC) of parasites was 3 × 10^6^/mL (or 2 × 10^6^/mL) in a final volume adjusted to 1 mL. The negative controls were parasites without treatment, and the positive control was treated with Bzn (5 µg/mL). According to Spina et al. [24], aliquots were collected at 24, 48, and 72 h, and they were suspended in 2% *p*-formaldehyde in PBS (0.15 M NaCl, 0.02 M NaH_2_PO_4_, and 0.017 M NaOH; pH 7.2). Then, the number of parasites (n° p) was counted in a Neubauer chamber. The percentage of inhibition was calculated as:

% inhibition = 100 − {[(n° p treated − InC)/(n° p control − InC)] × 100}

#### 2.5.3. Viability Assay

*T. cruzi* epimastigotes were incubated with 50 µg/mL of each BARE. Aliquots were taken at 24 and 48 h from each treated culture and were placed on slides for 3 min with 2% eosin in PBS (pH 7.2) and observed under a light microscope. The percentage of dead cells (stained) was determined for each treatment, which was carried out in triplicate. The percentage viability was calculated as:

% viability = [n° p alive/(n° p dead + n° p alive)] × 100

#### 2.5.4. Combination Assay (Synergism)

##### Drug Treatment

The IC_50_ value was first determined for each drug alone against *T. cruzi* epimastigotes (Dm28c strain). *H. hybrid* BARE concentrations ranged from 1 to 10 μg/mL for the single-drug treatment. Montanine concentrations ranged from 0.1 to 5 μg/mL. Bzn concentrations ranged from 1 to 5 μg/mL for the single-drug treatment. Combination studies were performed by combining montanine–*H. hybrid* BARE, montanine–Bzn, and *H. hybrid* BARE–Bzn. *T. cruzi* epimastigotes were treated with the drugs alone and combined in a fixed ratio [25] at concentrations of 0.25 × IC_50_, 0.5 × IC_50_, IC_50_, 2 × IC_50_, and 4 × IC_50_ (Figure S2). 

##### Analysis of Drug Interactions

To quantify drug interaction, the Combination Index (CI) and Dose Reduction Index (DRI) were assessed with the Chou and Talalay method [26] using the CompuSyn software (ComboSyn, Inc., New York, NY, USA). The mutually exclusive model was used, which is based on the assumption that drugs act through entirely different mechanisms [27]. CI was plotted on the y-axis as a function of the fraction affected (Fa) on the x-axis to assess drug synergism between drug combinations. Fa is a value between 0 and 1, where 0 means the drug had no effect on cell viability, and 1 means the drug produced a full effect on decreasing cell viability. The CI is a quantitative representation of pharmacological interactions. CI < 1 indicates synergism, CI = 1 indicates an additive interaction, and CI > 1 indicates antagonism. The DRI is a dimensionless measure of how much the dose of each drug in a synergistic combination may be reduced at a given fractional inhibition compared with the doses of each drug alone, where DRI > 1 indicates a favorable dose reduction, DRI < 1 indicates an unfavorable dose reduction, and finally, DRI = 1 indicates no dose reduction [26]. Experiments were conducted in triplicate. 

### 2.6. Statistical Analysis

Student’s *t*-test was used to determine the statistical significance of the differences between treated and control groups. The effect of each treatment was analyzed by one-way analysis of variance (ANOVA).

## 3. Results

### 3.1. GC-MS and UHPLC-MS/MS Analyses

Based on the total ion current (TIC) in the GC-MS analysis, the most abundant alkaloids identified in the Hippeastrum BAREs were montanine (*H. hybrid*); lycorine (*H. aglaiae*, *H. aulicum*, and *H. petiolatum*); hippeastrine (*H. puniceum*); 8-*O*-demethylhomolycorine (*H. reticulatum*); and tazettine (*H. glaucescens*). Further information regarding the alkaloid profile obtained by the GC-MS analysis of each BARE is shown in Table 1. The alkaloid chemical structures are shown in Figure S3.

The UHPLC-MS/MS quantification analysis showed a concentration of 14.32% montanine for the *H. hybrid* BARE, while the concentrations for *H. puniceum* and *H. aglaiae* BAREs were 1.28% and 4.98%, respectively. Finally, *H. petiolatum* showed the presence of montanine at a lower concentration (<1%). The linear correlation curve was r^2^ = 0.9202, and the linear equation was y = 113.638 x + 37,289. 

### 3.2. Cholinesterase Inhibitory Activities

The BAREs were tested for in vitro AChE and BuChE inhibitory activities. The results, expressed as IC_50_ values, are summarized in Table 2. Galanthamine was used as the positive control. All BAREs of *Hippeastrum* species showed strong activity against AChE. The most active BARE against AChE was *H. reticulatum*, followed by *H. petiolatum*, *H. puniceum*, and *H. aulicum* (IC_50_ = 3.13 ± 0.53, 5.07 ± 0.75, 5 ± 0.64, and 6.33 ± 0.81 μg/mL, respectively). Likewise, all BAREs showed moderate to low inhibitory activity against BuChE (IC_50_ ≥ 50 μg/mL).

### 3.3. Anti-T. cruzi Activity

#### 3.3.1. Growth Inhibition

##### Activity of Argentinean *Hippeastrum* spp. BAREs

The anti-*T. cruzi* activity of the BAREs in the epimastigote stage was evaluated at concentrations of 10 and 50 µg/mL. In Table 3, the results are shown. All *Hippeastrum* BAREs tested at 50 µg/mL presented 100% inhibition of proliferation at 24, 48, and 72 h, while at 10 µg/mL, the most active BAREs were *H. aglaiae* and *H. hybrid*, which inhibited 100% of the proliferation of epimastigotes at 72 h. Likewise, *H. aulicum*, *H. glaucescens*, *H. petiolatum*, *H. puniceum*, and *H. reticulatum* showed the potent inhibition of epimastigote proliferation at 48 h. Bzn presented strong proliferation inhibition, with a slight loss of efficacy throughout the assay.

##### Antiproliferative *T. cruzi* Activity of *H. aglaiae* and *H. hybrid* BAREs

**Figure 1:**
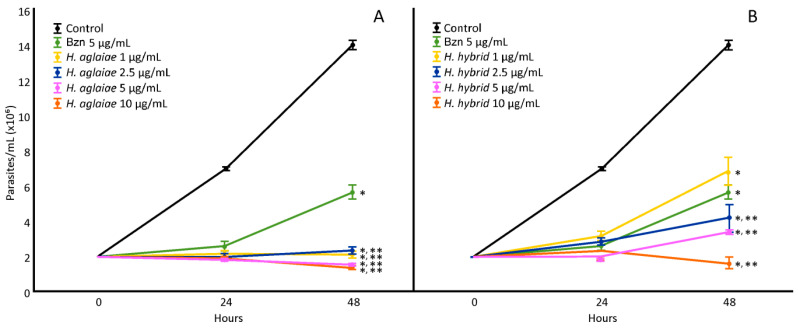
Effect of the *H. aglaiae* BARE (**A**) and *H. hybrid* BARE (**B**) on *T. cruzi* epimastigote proliferation at concentrations of 1, 2.5, 5, and 10 µg/mL. *: Significant difference from negative control; **: significant difference from positive control. *p* < 0.05.

*H. aglaiae* and *H. hybrid* BAREs were evaluated (1, 2.5, 5, and 10 µg/mL) in order to determine the IC_50_ values (Figure 1). The *H. aglaiae* BARE showed 100% inhibition of the proliferation of *T. cruzi* at 48 h at all concentrations tested. Since all concentrations showed the potent inhibition of epimastigote proliferation, no dose-dependent relationship was observed. Regarding the *H. hybrid* BARE, all of the concentrations assayed (2.5, 5, and 10 µg/mL) reduced the number of parasites compared to the control. These results indicate an effect on proliferation greater than that of Bzn (positive control). Thus, the IC_50_ values for the *H. aglaiae* and *H. hybrid* BAREs were 0.0026 and 0.96 µg/mL, respectively, which are lower than that of Bzn (4.58 µg/mL).

##### Anti-*T. cruzi* Activity of *H. hybrid* BARE Fractions

**Figure 2:**
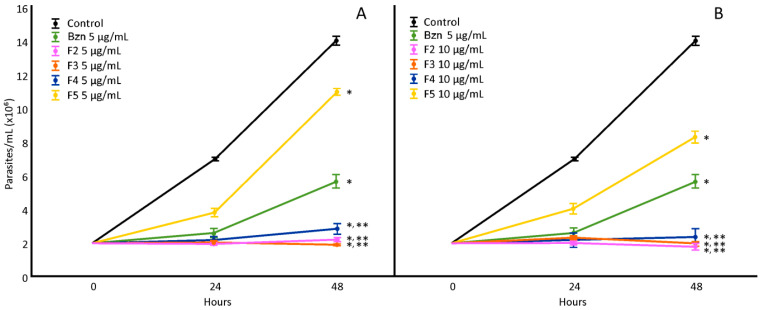
Effect of *H. hybrid* BARE fractions on *T. cruzi* epimastigotes at concentrations of 5 µg/mL (**A**) and 10 µg/mL (**B**). *: Significant difference from negative control; **: significant difference from positive control. *p* < 0.05.

The *H. hybrid* BARE fractions (F1–F6) were tested at 5 and 10 µg/mL (Figure 2), showing a notable decrease in proliferation compared to the untreated control for fractions F2, F3, F4, and F5 (F1 and F6 did not show differences; data not shown). F3 was the most active at 5 µg/mL, and F2 was the most active at 10 µg/mL. Through UHPLC-MS/MS analysis, fraction F2 revealed mainly the presence of montanine (13.82%), while F3 showed almost double the amount of this alkaloid (24.76%) and also hippeastrine. F4 showed a mixture of 7-hydroxyclivonine, hippeastrine, and montanine (10.20%). Finally, in F5, the fragmentation patterns indicated the presence of 7-hydroxyclivonine and a low concentration of montanine (<1%). 

##### Antiproliferative Activity of Montanine

**Figure 3:**
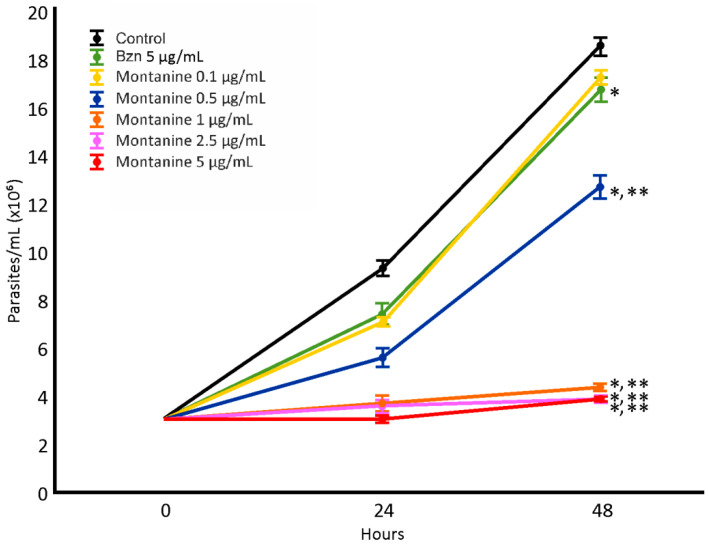
Effect of montanine in *T. cruzi* epimastigotes. *: Significant difference from negative control; **: significant difference from positive control. *p* < 0.05.

The epimastigote antiproliferative assay was performed with montanine at concentrations of 0.1, 0.5, 1, 2.5, and 5 µg/mL (Figure 3). Potent dose-dependent activity was observed, and all concentrations tested (except 0.1 µg/mL) were more active than Bzn. The IC_50_ value for montanine was 0.55 µg/mL. 

#### 3.3.2. Viability Assay

The viability percentages of epimastigote treated with *Hippeastrum* BAREs at 50 µg/mL and Bzn at 5 µg/mL for 24 and 48 h are shown in Table 4. All *Hippeastrum* BAREs produced a decrease in the viability of *T. cruzi* epimastigotes, with the *H. reticulatum* and *H. hybrid* BAREs being those with the greatest effect in the first 24 h (87.84 and 86.59%, respectively). Likewise, the *H. glaucescens* BARE decreased the viability of *T. cruzi* epimastigotes to a value of 72.88% at 48 h. Regarding Bzn, the viability of epimastigotes was not affected, presenting approximately 98% living cells.

#### 3.3.3. Synergistic Combinations

The growth inhibition assay was performed for each drug alone against *T. cruzi* epimastigotes, and the CompuSyn software was used for the generation of single-drug dose–effect curves (Figure S4A). The combinations of montanine + *H. hybrid* BARE, montanine + Bzn, and *H. hybrid* BARE + Bzn were evaluated against *T. cruzi* (Figures S4B and S5). 

**Figure 4:**
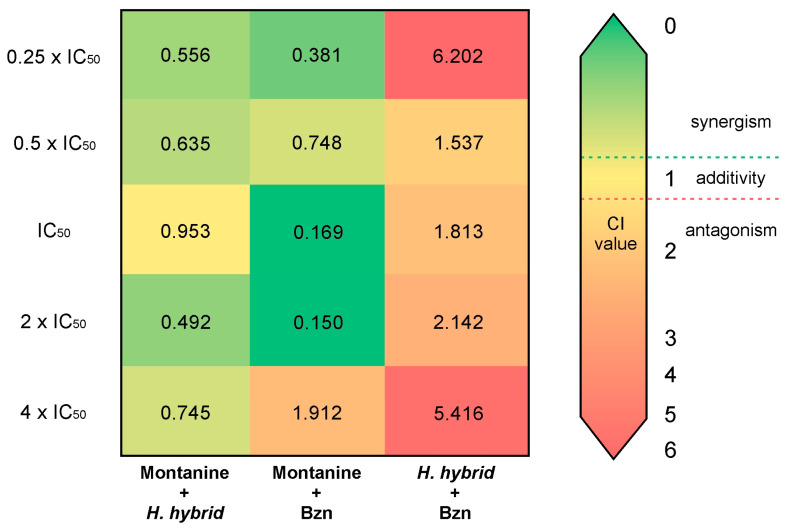
Synergy heat map compiling CI values. The squares in the figure indicate antagonism (CI > 1.2, orange-red), additivity (0.9 < CI < 1.1, yellow), and synergism (CI < 0.9, green) among montanine, *H. hybrid* BARE, and Bzn against *T. cruzi* epimastigotes.

The results of the combinations are summarized in Table S1. The combination of montanine + Bzn shows the best synergistic effect, particularly in the mixtures of IC_50_ and 2 × IC_50_ (CI = 0.169 and 0.150, respectively). This interaction also demonstrated a potent effect on the proliferation of *T. cruzi* epimastigotes, with Fa values = 0.972 and 0.99, respectively. A combination of lower concentrations of montanine + Bzn also resulted in a synergistic effect (CI < 1) with a Fa of nearly 0.7. However, the combination of a higher concentration of montanine + Bzn (4 × IC_50_) resulted in an antagonistic effect (CI = 1.912). Likewise, all combinations of montanine + *H. hybrid* BARE showed synergistic interactions, with the combination of 2 × IC_50_ (montanine = 1.1 µg/mL + *H. hybrid* BARE = 1.24 µg/mL) being the one with the highest synergistic effect (CI = 0.492) and that of 4 × IC_50_ being the one with the highest inhibitory effect on the proliferation of *T. cruzi* (Fa = 0.972). However, *H. hybrid* BARE + Bzn showed antagonistic effects in all combinations (CI = 1.537–6.202) and low to moderate epimastigote antiproliferative effects (Fa = 0.05–0.708). The CI values of all combinations are shown in Figure 4. Based on the DRI values of the actual experimental data points, all synergistic drug combinations achieved favorable dose reduction indices (DRI > 1) for both drugs involved (Table S1). Montanine + Bzn reached the highest value of the DRI at a concentration of 2 × IC_50_, reducing the dose of montanine by 9.9 times and the dose of Bzn by 20.19 times to obtain 99% inhibition of the proliferation of *T. cruzi*. These interesting results were obtained by combining montanine + Bzn at their IC_50_ concentration values, achieving an inhibition value of 97% for both compounds and dose reductions of 9.4 and 15.8 times for montanine and Bzn, respectively. Finally, for all montanine + *H. hybrid* BARE combinations, favorable DRI values were obtained (1.63 to 5.57). 

## 4. Discussion

The chemical profiles of the *Hippeastrum* species BAREs showed a total of fifteen alkaloids. This is the first report of the presence of 11,12-dehydroanhydrolycorine in *H. aulicum*, 8-*O*-demethylhomolycorine, hippeastrine, and montanine in *H. puniceum*, and 8-*O*-demethylhomolycorine in *H. reticulatum*, expanding the list of alkaloids previously reported for these species [28,29,30]. This work presents the first alkaloid profiles of *H. aglaiae*, which shows the presence of the alkaloids 8-*O*-demethylhomolycorine, lycorine, montanine, and norlycoramine, and of *H. petiolatum*, which shows the presence of montanine and lycorine. In the *H. hybrid* BARE, the alkaloids 2-hydroxyhomolycorine, 7-hydroxyclivonine, galanthamine, hippeastrine, lycorine, montanine, and pancracine were identified. 

The alkaloids pancracine, montanine, lycorine, hamayne, 8-*O*-demethylhomolycorine, hippeastrine, and norlycoramine identified in the *Hippeastrum* BAREs have previously been tested against AChE and BuChE, reporting low or null inhibitory activities [11,16,31,32,33]. Herein, the *H. reticulatum* BARE exhibited the highest inhibitory activity against both AChE (IC_50_ = 3 ± 0.53) and BuChE (IC_50_ = 50 ± 1.20). The alkaloids lycorine, 11,12-dehydroanhydrolycorine, and 8-*O*-demetylhomolycorine and three unidentified narcissidine-type alkaloids were identified in this BARE, suggesting that the inhibition could be due to the combined effect of these compounds or other alkaloids present in low abundance.

Regarding anti-*T. cruzi* activity, the alkaloids identified in the BAREs, lycorine, hippeastrine, and montanine, have been reported for their inhibitory effect against the amastigote form [34], whereas pancracine has shown activity against trypomastigotes of *T. cruzi* [35]. Recently, de Souza and Barrias [36] proposed a new scheme of the life cycle of *T. cruzi*, describing epimastigote-type forms with infective and proliferative capacity, highlighting the importance of taking into account these stages (epimastigote-type forms), and establishing that it is a new target for treatment during the course of Chagas disease. This new approach supports the previous work developed by Kessler et al. [37], who reported differentiated epimastigotes with the ability to infect mammalian cells. Based on these advances in CD research, the *Hippeastrum* BAREs were assayed on *T. cruzi* epimastigotes.

In the literature, the IC_50_ values of Amaryllidaceae alkaloid extracts have been reported for their anti-*T. cruzi* activity when tested at ≥100 µg/mL [38,39]. The *H. aglaiae* and *H. hybrid* BAREs showed the highest effect against the proliferation of *T. cruzi* epimastigotes, with IC_50_ values lower than that of Bzn (IC_50_ = 0.0026 µg/mL, IC_50_ = 0.96 µg/mL, and IC_50_ = 4.58 µg/mL, respectively). In addition, the *H. aglaiae* and *H. hybrid* BAREs’ antiproliferative activity persisted even at 72 h. The *H. aglaiae* and *H. hybrid* BARE activities could be explained by the presence of lycorine and montanine, respectively. In order to prove the compound responsible for the *H. hybrid* BARE activity and its fractions, the assay of pure montanine was evaluated against *T. cruzi*, showing a dose-dependent response.

Combined therapies are used to treat various infectious diseases, including toxoplasmosis, malaria, tuberculosis, and AIDS [1]. Likewise, therapies for the treatment of CD aim to reduce the side effects produced by Bzn [7,40]. Some alkaloids, such as carbol, have been tested in combination with Bzn, reducing parasitemia in murine models [41]. Herein, the synergistic effect of the combination of montanine with Bzn stands out, managing to reduce the dose of Bzn by around 20 times. In addition, montanine is reported to have a low rate of cytotoxicity in HepG2 cells, with a value of TC_50_ = 13.9 µg/mL (46.10 µM) [34]. This is the first study showing that montanine and Bzn, as a combined strategy, have a powerful inhibitory effect against *T. cruzi* forms. These results position the montanine alkaloid as an enhancer of the effect of Bzn, reducing the doses used to achieve inhibition close to 100%. Also interesting is the synergistic effect produced by adding montanine to the *H. hybrid* BARE. However, the combination of the *H. hybrid* BARE and Bzn showed an antagonistic effect. These results indicate that the combination of several compounds reported as active does not always imply greater activity. In this case, the alkaloids present in the BARE reduced the effect of Bzn.

Possibly, the pool of alkaloids present in *H. hybrid* with the extra addition of montanine enhances the antiparasitic activity, leaving a window for future trials on the effect of the interaction between alkaloids of the Amaryllidaceae family against *T. cruzi*.

## 5. Conclusions

The combination of the montanine alkaloid and Bzn showed a potent synergistic effect, reducing the concentration of Bzn twenty-fold against *T. cruzi*, and could be considered for future research to reduce the side effects of Bzn to treat CD. To a lesser extent, montanine and the *H. hybrid* BARE presented a synergistic effect. Thus, further mechanistic studies, such as the determination of the structure–activity relationship, must be performed to identify the potential compounds responsible for these properties. Finally, Argentinean *Hippeastrum* species represent a promising candidate for the treatment of CD.

## Figures and Tables

**Table 1:** Main alkaloids identified in Argentinean *Hippeastrum* spp. samples by GC-MS analysis.

Alkaloids	RI	[M^+^]	MS	Species
Galanthamine (1)	21.322	287	286 (100), 270 (13), 244 (24), 230 (12), 216 (33), 174 (27), 115 (12)	*H. hybrid*
Norlycoramine (2)	22.390	275	274 (100), 202 (10), 188 (12), 178 (5)	*H. aglaiae*
11,12-Dehydroanhydrolycorine (3)	24.796	249	248 (100), 191 (10), 190 (24), 189 (6), 164 (3), 163 (7), 123 (6), 95 (14)	*H. aulicum* *H. reticulatum*
Montanine (4)	24.916	301	270 (87), 257 (37), 252 (24), 229 (26), 226 (30), 223 (29), 199 (20), 185 (32), 115 (21)	*H. aglaiae*, *H. hybrid* *H. petiolatum* *H. puniceum*
Tazettine (5)	25.313	331	298 (19), 248 (15), 247 (100), 201 (16), 199 (14), 181 (13), 115 (15), 71 (15), 70 (17)	*H. glaucescens*
*m*/*z* 264 (NP Narcissidine type) (6)	25.592	265	264 (100), 178 (13), 89 (10), 103 (10), 266 (10), 206 (9), 75 (6), 150 (6), 177 (6)	*H. reticulatum*
Pancracine (7)	26.070	287	286 (23), 270 (19), 243 (25), 223 (27), 214 (24), 199 (32), 185 (42), 128 (21), 115 (24)	*H. hybrid*
Hamayne (8)	26.206	287	259 (15), 258 (100), 242 (10), 211 (13), 186 (15), 181 (17), 128 (14), 115 (13)	*H. aulicum*
Lycorine (9)	26.759	287	286 (19), 268 (24), 250 (15), 227 (79), 226 (100), 211 (7), 147 (15)	*H. aglaiae*, *H. aulicum* *H. hybrid* *H. petiolatum* *H. reticulatum*
8-*O*-Demetylhomolycorine (10)	27.528	301	109 (100), 108 (22), 110 (8), 82 (3), 94 (3), 93 (2), 65 (2)	*H. aglaiae* *H. puniceum* *H. reticulatum*
Hippeastrine (11)	28.620	315	96 (40), 125 (100), 315 (<1)	*H. hybrid* *H. puniceum*
2-Hydroxyhomolycorine (12)	29.223	331	125 (100), 95 (3), 65 (2), 42 (10)	*H. hybrid*
*m*/*z* 294 (NP Narcissidine type) (13)	29.294	295	294 (100), 165 (16), 296 (15), 152 (10), 252 (8), 135 (8), 253 (7), 166 (6), 238 (4)	*H. reticulatum*
*m*/*z* 280 (NP Narcissidine type) (14)	29.805	281	280 (100), 282 (10), 152 (5), 253 (4), 252 (3), 238 (3), 151 (3), 266 (2), 103 (2)	*H. reticulatum*
7-Hydroxyclivonine (15)	29.857	333	178 (3), 97 (5), 96 (64), 84 (5), 83 (100), 82 (32), 44 (3), 42 (8)	*H. hybrid*

**Table 2:** Cholinesterase inhibitory activities of Argentinean *Hippeastrum* spp. BAREs.

Species	IC_50_ (μg/mL) of BAREs	
	AChE	BuChE
*H. aglaiae*	16.68 ± 0.98	80.66 ± 1.40
*H. aulicum*	6.33 ± 0.81	>100
*H. glaucescens*	9.08 ± 0.82	>100
*H. hybrid*	15.42 ± 1.11	>100
*H. petiolatum*	5.35 ± 0.75	98.07 ± 1.30
*H. puniceum*	5.07 ± 0.64	95.39 ± 1.59
*H. reticulatum*	3.13 ± 0.53	50.05 ± 1.2
Galanthamine ^a^	0.16 ± 0.05	5.82 ± 0.34

^a^ Reference compound.

**Table 3:** In vitro activity of *Hippeastrum* spp. BAREs from Argentina on proliferation of *T. cruzi* epimastigotes (mean ± SD).

Species	Growth Inhibition (%)					
	10 µg/mL			50 µg/mL		
	24 h	48 h	72 h	24 h	48 h	72 h
*H. aglaiae*	100	100	100	100	100	100
*H. aulicum*	100	100	95.89 ± 1.45	100	100	100
*H. glaucescens*	69.83 ± 2.25	100	47.3 ± 1.88	100	100	100
*H. hybrid*	91.38 ± 0.63	100	100	100	100	100
*H. petiolatum*	100	73.96 ± 1.67	35.03 ± 3.38	100	100	100
*H. puniceum*	95.69 ± 0.42	100	57.03 ± 2.23	100	100	100
*H. reticulatum*	100	100	38.12 ± 4.77	100	100	100
Bzn *	100	98.33 ± 0.34	95.68 ± 1.48	100	98.33 ± 0.34	95.68 ± 1.48

* The concentration of Bzn was 5 µg/mL.

**Table 4:** In vitro activity of *Hippeastrum* spp. BAREs from Argentina on viability of *T. cruzi* epimastigotes (mean ± SD).

Species	Viability (%)	
	50 µg/mL	
	24 h	48 h
*H. aglaiae*	92.03 ± 0.95	84.78 ± 3.36
*H. aulicum*	88.61 ± 1.73	83.48 ± 1.08
*H. glaucescens*	88.18 ± 2.38	72.88 ± 0.88
*H. hybrid*	86.59 ± 0.19	82.54 ± 1.35
*H. petiolatum*	88.25 ± 1.97	86.12 ± 4.19
*H. puniceum*	89.02 ± 2.36	80.43 ± 2.78
*H. reticulatum*	87.84 ± 1.77	82.65 ± 3.55
Bzn *	99.33 ± 0.02	97.06 ± 0.37

* The concentration of Bzn was 5 µg/mL.

## Data Availability

The data presented in this study are available on request from the corresponding author.

## Supplementary Materials

The following supporting information can be downloaded at: https://www.mdpi.com/article/10.3390/microorganisms11010144/s1. Figure S1: GC-MS chromatograms of *Hippeastrum* BAREs a: *H. aglaiae*; b: *H. aulicum*; c: *H. glaucescens*; d: *H. hybrid*; e: *H. petiolatum*; f: *H. puniceum*; g: *H. reticulatum*. Figure S2: Representation of the design of the combination experiences; Figure S3: Alkaloids identified in *Hippeastrum* BAREs; Figure S4: The dose-effect curves of single drugs (A) and drug combos (B); Figure S5. Chou-Talalay method Fa-CI plot of montanine, *H. hybrid* BARE, and Bzn. Table S1: CI, DRI and Fa values of combinations of montanine, *H. hybrid* BARE and Bzn.
