# Supplementary material for: Antiparasitic Activity of Hippeastrum Species and Synergistic Interaction between Montanine and Benznidazole against Trypanosoma cruzi
Source: Microorganisms. 2023 Jan 6;11(1):144. doi: 10.3390/microorganisms11010144 (PMC9864487; doi:10.3390/microorganisms11010144)
Supplement: Supplementary file 1 [file microorganisms-11-00144-s001.zip › microorganisms-2146597-supplementary.pdf]

Supplementary material

Antiparasitic Activity of *Hippeastrum* Species and Synergistic Interaction between Montanine and Benznidazole against *Trypanosoma cruzi*

Mauricio Piñeiro [1,2,†], Javier E. Ortiz [1,2,†], Renata M. Spina Zapata [3], Patricia A. Barrera [3], Miguel A. Sosa [3], Germán Roitman [4], Jaume Bastida [5] and Gabriela E. Feresin [1,2,*]

[1] Instituto de Biotecnología, Facultad de Ingeniería, Universidad Nacional de San Juan, Av. Libertador General San Martin 1109 O, San Juan CP 5400, Argentina
[2] Consejo Nacional de Investigaciones Científicas y Técnicas (CONICET), Ciudad Autónoma de Buenos Aires (CABA), Godoy Cruz CP 2290, Argentina
[3] Facultad de Ciencias Médicas, Instituto de Histología y Embriología "Dr. Mario H. Burgos", Universidad Nacional de Cuyo-CONICET, Mendoza CP 5500, Argentina
[4] Facultad de Turismo y Urbanismo, Universidad Nacional de San Luis, Av. del Libertador San Martín 721 Villa de Merlo, San Luis CP D5881DFN, Argentina
[5] Departament de Biologia, Sanitat i Medi Ambient, Facultat de Farmàcia i Ciències de l'Alimentació, Universitat de Barcelona, 08028 Barcelona, Spain
* Correspondence: gferesin@unsj.edu.ar
† These authors contributed equally to this work.

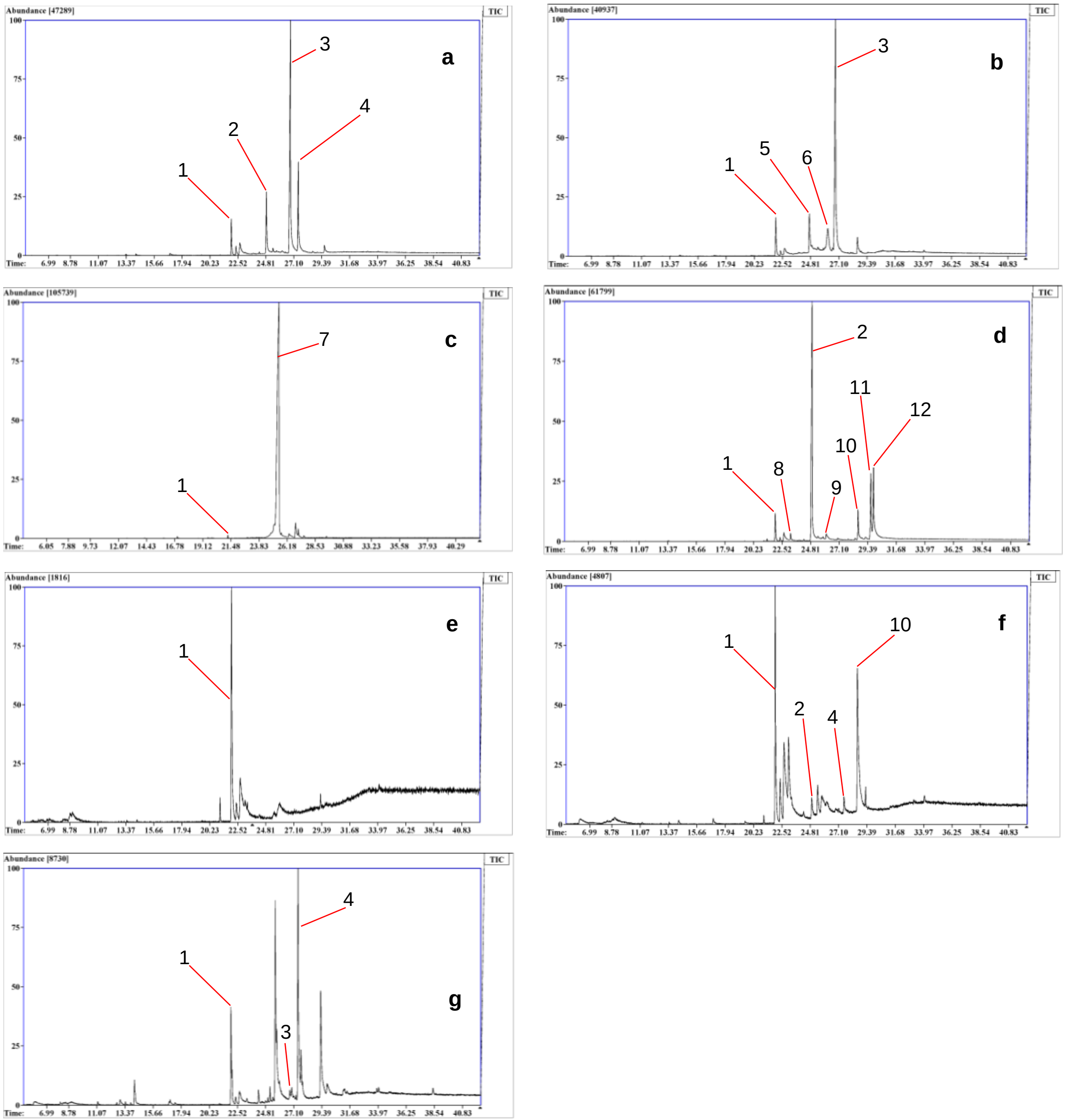

Figure S1. GC-MS chromatograms of *Hippeastrum* BAREs

a: *H. aglaiae*; b: *H. aulicum*; c: *H. glaucescens*; d: *H. hybrid*; e: *H. petiolatum*; f: *H. puniceum*; g: *H. reticulatum*. The signals related to the identified alkaloids are indicated on each chromatogram: (1) codeine, (2) montanine, (3) lycorine, (4) 8-*O*-demethylhomolycorine, (5) 11,12-dehydroanhydrolycorine, (6) hamayne, (7) tazettine, (8) 4-*O*-methylnangustine, (9) pancracine, (10) hippeastrine, (11) 2-OH-homolycorine, (12) 7-OH-clivonine. The alkaloids galantamine and norlycoramine were detected in low amounts in *H. hybrid* and *H. aglaiae,* respectively.

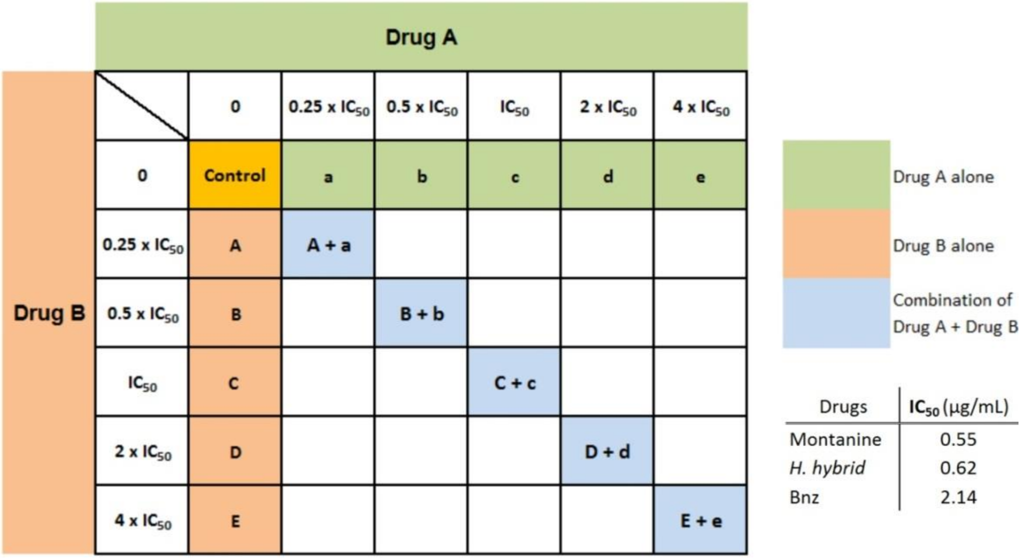

Figure S2. Representation of the design of the combination experiences.

T. cruzi epimastigotes were treated with Drug A and Drug B alone and in combination in a fixed ration for 48 h, in the concentrations of 0.25×IC_{50},0.5×IC_{50}, IC_{50}, 2×IC_{50}, and 4×IC_{50}.

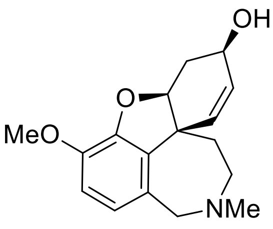

Galanthamine

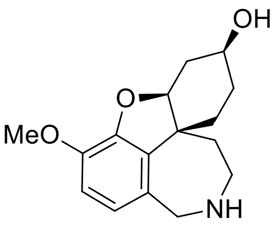

Norlycoramine

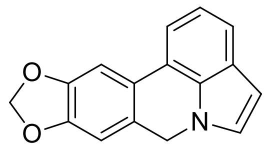

11,12-dehydroanhydrolycorine

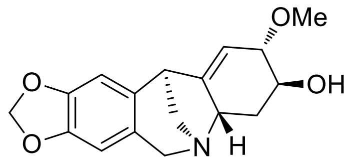

Montanine

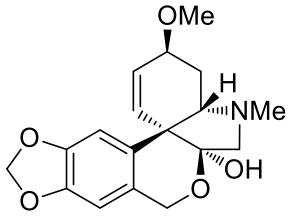

Tazettine

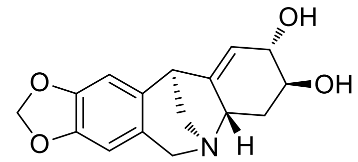

Pancracine

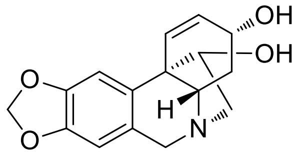

Hamayne

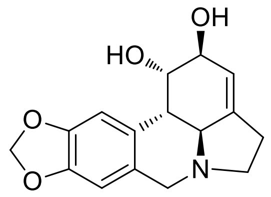

Lycorine

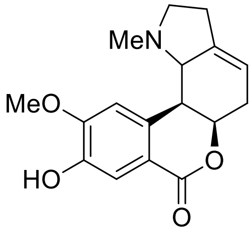

8-O-Demethylhomolycorine

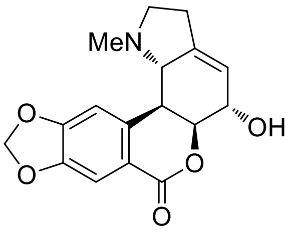

Hippeastrine

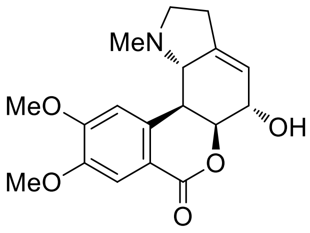

2-hydroxyhomolycorine

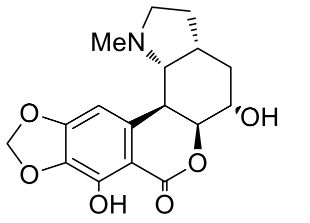

7-hydroxyclivonine

Figure S3. Alkaloids identified in *Hippeastrum* BAREs.

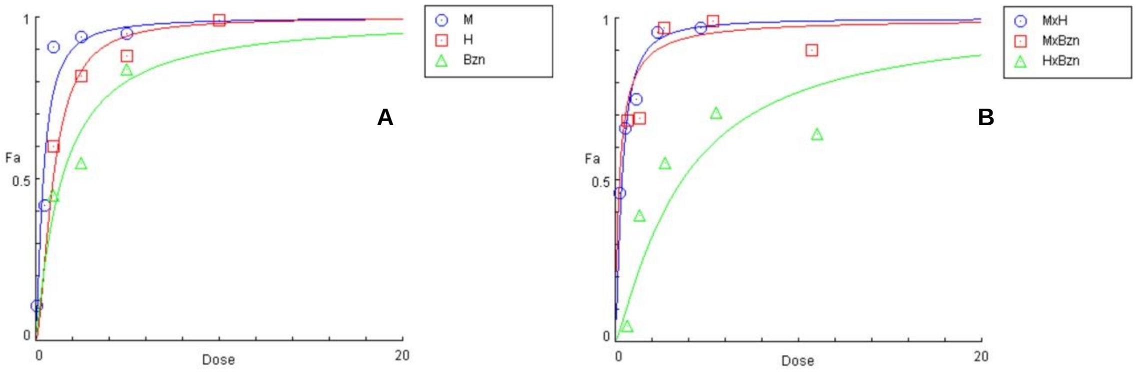

Figure S4. The dose-effect curves of single drugs (A) and drug combos (B).

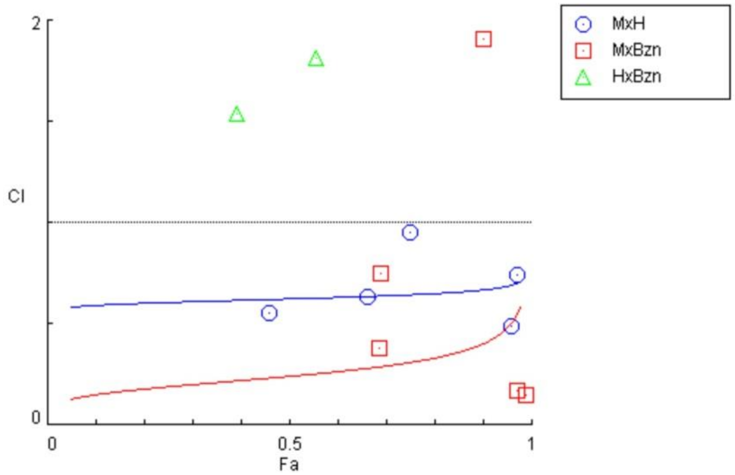

Figure S5. Chou-Talalay method Fa-CI plot of montanine, *H. hybrid* BARE, and Bzn.

CI was plotted on y-axis as a function of fractional effect (Fa) on the x-axis to assess drug synergism. CI<1, CI=1 and CI>1 indicate synergism, additivity, and antagonism, respectively.

Table S1. CI, DRI and Fa values of combinations of montanine, *H. hybrid* BARE and Bzn.

Combination (Drug A + Drug B)		Concentration (µg Drug A + µg Drug B)	Fraction Affected (Fa)	CI	DRI	
Montanine + *H. hybrid* BARE	0.25 x IC_{50}	0.137 + 0.155	0.460	0.557	M = 2.65	H = 5.57
	0.5 x IC_{50}	0.275 + 0.309	0.662	0.635	M = 2.40	H = 4.55
	IC_{50}	0.550 + 0.619	0.750	0.954	M = 1.63	H = 2.93
	2 x IC_{50}	1.100 + 1.239	0.958	0.493	M = 3.47	H = 4.87
	4 x IC_{50}	2.200 + 2.479	0.972	0.746	M = 2.34	H = 3.12
Montanine + Bzn	0.25 x IC_{50}	0.137 + 0.534	0.685	0.382	M = 5.18	B = 5.30
	0.5 x IC_{50}	0.275 + 1.069	0.690	0.749	M = 2.63	B = 2.70
	IC_{50}	0.550 + 2.139	0.972	0.170	M = 9.38	B = 15.81
	2 x IC_{50}	1.100 + 4.279	0.990	0.150	M = 9.93	B = 20.19
	4 x IC_{50}	2.200 + 8.558	0.903	1.913	M = 0.91	B = 1.21
H. hybrid BARE + Bzn	0.25 x IC_{50}	0.155 + 0.534	0.050	6.202	H = 1.07	B = 0.18
	0.5 x IC_{50}	0.309 + 1.069	0.393	1.538	H = 2.36	B = 0.89
	IC_{50}	0.619 + 2.139	0.555	1.814	H = 1.74	B = 0.80
	2 x IC_{50}	1.239 + 4.279	0.708	2.142	H = 1.29	B = 0.73
	4 x IC_{50}	2.479 + 8.558	0.643	5.416	H = 0.54	B = 0.27

CI in red, indicate concentrations of drug pairs that are synergic.
